# Contributions of biological tests and the 4 Ts score in the diagnosis of Heparin Induced Thrombocytopenia

**Published:** 2012-09-12

**Authors:** Khalil Haouach, Brahim Admou, Pascal Lauriant, Laila Chabaa

**Affiliations:** 1 Laboratory of hematology, Faculty of Medicine, Cadi Ayyad University, University Hospital, Marrakech, Morocco

**Keywords:** Heparin-induced thrombocytopenia, antibodies, 4 Ts scoring system

## Abstract

**Introduction:**

Heparin-induced thrombocytopenia (HIT) is an adverse drug reaction caused by antibodies to the heparin/platelet factor 4 (PF4) complexes. HIT diagnosis is challenging and depends on clinical presentation and laboratory tests. We investigated the interest of the combined use of 4 Ts score and the functional and immunological tests for the diagnosis of HIT.

**Methods:**

We analyzed 178 patients with suspected HIT, for which the 4 Ts score was calculated. Heparin-PF4 antibodies were detected by both Heparin-induced platelet activation test (HIPA) and Heparin platelet induced antibodies enzyme immunoassay.

**Results:**

Our results shown that in low probability group, 85% of plasmas were found negative versus 55.5% in the high probability group. On the other hand, 22.2% of patients were HIT positive in high probability group versus 0% in the low probability group.

**Conclusion:**

These results confirmed that the negative predictive value of the HIT score was high. The 4T's model has demonstrated excellent sensitivity but its specificity was limited. The specificity of the functional and immunological test is high only in a context suggestive of HIT. Both methods should be considered complementary in the diagnostic strategy.

## Introduction

Heparin-induced thrombocytopenia (HIT) is a prothrombotic adverse drug reaction caused by heparin [1]. HIT complicates 0.5% to 5% long-term (7 to 14 days) treatment by unfractionated heparin (UFH) [2–4]. Is a peripheral thrombocytopenia due to the appearance of antibodies against a macromolecular complex heparin / platelet factor 4 (F4P) [5]. These antibodies have the potential to activate platelets, endothelial cells, and monocytes, leading to a systemic activation of coagulation responsible for the thrombotic symptoms often associated with this syndrome [6].

The diagnosis of HIT relies upon a combination of clinical assessment and laboratory testing. Recently, Warkentin and Heddle developed a new scoring system for the pretest probability of HIT: the 4 T's scoring system (Table 1) [7], which takes advantage of new information regarding the clinical features of HIT and is simple to apply prospectively. Two categories of assays are available for laboratory detection of HIT antibodies: functional assays and immunological assays [8, 9].

**Table 1:** The 4 T's scoring system

Characteristics	Points	
Thrombocytopenia	2	> 50% platelet fall or nadir ≥ 20 G/l
	1	30-50% platelet fall or nadir10-19 G/l
	0	<30% platelet fall or nadir < 10 G/l
Timing of platelet count fall	2	Clear onset between days 5–10 or ≤1 day (if heparin exposure within past 30 days)
	1	Consistent with day's 5–10 fall, but not clear (missing platelet counts) or ≤1 day (heparin exposure within past 31–100 days)
	0	Platelet count fall ≤4 days without recent heparin exposure
Thrombosis or other squeal	2	New thrombosis; skin necrosis; acute systemic reaction after intravenous heparin bolus
	1	Progressive or recurrent thrombosis; erythematous skin lesions; suspected thrombosis (not yet proven)
	0	None
other cause ofthrombocytopenia	2	Noneevident
	1	Possible
	0	Defined

Probabilityof HITbefore testingaccording to thetotal score. 6-8: High, 4-5: Intermediate, 0-3: low

Immunological assays are easy to perform; they are well standardized and accessible to all laboratories. Their sensitivity is excellent, but their specificity remains disappointing, especially in contexts of postoperative cardiopulmonary bypass and in several specific clinical context (pregnancy, diabetes, antiphospholipid syndrome, lupus) [8, 10].

Functional assays are more specific for the diagnosis of HIT. However, they are time-consuming (platelet aggregation tests), involving the collection of platelets from healthy controls, which is or not feasible in every laboratory since it requires the use of radioisotopes [10–12].

HIT must be identified as early possible, because the lack of appropriate and early care may present a risk of dramatic complications and life-consequences. Given the limitations of each of the biological tests available, none is completely satisfactory. It's important for each specialized laboratories to develop a diagnostic strategy, based on the association of clinical assessment and several methods for detection to heparin-PF4 antibodies.

The aim of our study was to evaluate the efficacy in a single-centre of the combined use of the 4 Ts score and the functional and immunological tests for the diagnostic strategy of HIT.

## Methods

### Patients and methods

Our study included the patients evaluated for suspected HIT. The evaluations were conducted prospectively by hematologist clinician who estimated the pretest probability of HIT, using the 4T's scoring system as previously described. Complete clinical and laboratory data was available for 178 of 185 patients referred for HIT testing between January 2010 and June 2011. The remaining 7 patients did not have a clinical score assigned and were thus excluded from further analysis.

The median age was 67 (range 2-87 years) and 113 (63.5%) were male. Our series included 12 children (6.7%). Mean platelet count nadir was 81 G/L (4- 421 G/L) and thrombocytopenia (platelet count < 100G/L) was present in 119 patients (66.9%). All patients had been treated with UFH (50.6%), low-molecular-weight heparin (39.3%) or both (10.1%).

### Laboratory testing for HIT antibodies

Citrated (0.129 mol/ L) plasma samples were obtained from all patients. Platelet free plasma was prepared by double centrifugation at 2500 g for 15 min at room temperature and was stored at - 80°C before assay. Heparin-PF4 antibodies were detected by both functional and two types of antigenic assays.

**Heparin-induced platelet activation assay (HIPA):** The functional assay used for detection of platelet-activating antibodies was heparin-induced platelet activation. The patient′s samples were incubated in the presence of heparin and platelet control, obtained from citrated platelet-rich plasma from three normal donors. Concentrations of heparin were similar to those used in therapy. HIPA test was considered positive if at least three donors platelets showed activation in the presence of a low (0.5 et 1 IU/ml) but not a high (100 IU/mL) heparin concentration, with a lag time of 30 minutes or less and all the control wells reacting as expected. Weakly-reacting sera (30-45 minutes lag time) were considered negative.

**Heparin platelet induced antibodies (HPIA) enzyme immunoassay:** The immunoassay used was a commercial PF4/heparin enzyme-linked immunoabsorbent test (ELISA) kit according to the manufacturer's instructions (HPIA IgGAM), this test detects anti-PF4/heparin antibodies of all three major immunoglobulin (Ig) classes, IgG, IgM, and IgA, with a cutoff of 0.5 optical density (OD) units.

**Detection of antibodies by the particle gel immunoassay ID-heparin/PF4 PaGIA:** The particle test gel immunoassay ID-heparin/PF4 PaGIA was performed on fresh citrated plasma according to the manufacturer's protocol.

### Diagnostic strategy

The research of anti-F4P potentially responsible of HIT was performed when: Platelet count <100 G/L, and/or a relative fall of platelet counts over two consecutive counts(over 30%) during treatment by heparin; a venous or arterial thrombosis during treatment with heparin

HIPA and immunoassays were performed on samples from patients with intermediate or high pre-test probability. If the pre-test probability score was low (<3), the diagnostic strategy was based firstly on achieving the test ID-heparin/PF4 PaGIA, that is a rapid test enabling render result in less than 1 hour. This test is strictly qualitative, it allows, if negative, to exclude the diagnosis of HIT (negative predictive value approaching 100%) [12], and therefore continuing treatment with heparin. When the result was positive or doubtful, diagnostic evidence was provided by the realization of two quantitative techniques for which cut-off points are established, enabling an adequate therapeutic decision. If the pre-test probability score was high or intermediate, therapeutic decision making by physicians was to stop heparin and substitute it by another antithrombotic as soon that there is a clinical suspicion of HIT. The samples of these patients were then stored and analyzed in series using both confirmation techniques previously described, HPIA and HIPA assays. In combining these two methods, negative predictive value can reach 100% [13].

A positive result or no clear agglutination was confirmed by HIPA and immunoassays. A sample was considered positive if positive results were obtained with both HIPA and immunoassays. Results were defined as indeterminate if only one test, HIPA or immunoassays, was positive. Patients were classified as negative if both HIPA (tested with three platelet donors) and immunoassays had negative results.

## Results

Of 178 patient plasmas, 12 (6.7%) tested positive for HIT antibodies in the HIPA test and in the PF4/Heparin ELISA IgGAM, and were considered as HIT positive. 139 patient plasma (78.1%) were found negative with both methods. In our series, we obtained an undetermined diagnosis for 27 patients (15.2%), 21 (11.8%) of whom were ELISA positive and 6 (3.4%) of whom were HIPA positive (Table 2).

**Table 2:** Results of the immunological and functional test

Patients	Patients HIT +	Patients HIT -	Patients undetermined HIT	
Results of essays	HIPA+ /ELISA +	HIPA-/ELISA-	HIPA-/ELISA +	HIPA + /ELISA-
Total	12 (6.7%)	139(78.1%)	21 (11.8%)	6 (3.4%)
			27 (15.2)	

Table 3 summarizes the results of different pathologies identified according to the 4T's score. 40 of 178 patients (22.5%) were classified as having a low risk for HIT, 129 (72.5%) have a intermediate risk, and only patients 9 (5.1%) have a high risk for HIT.

**Table 3:** Heparin-induced thrombocytopenia pretest probability categories and patient types

Pre-test category	Internal medicine	General surgery	Cardiovascular surgery	Intensive Care	Neurology/neurosurgery	Orthopedic surgery	Total
Low	9	8	7	11	4	1	40
Intermediate	54	22	12	31	4	6	129
High	2	1	2	2	1	1	9
Total	65 (36.5%)	31 (17.4%)	21 (11.8%)	44 (24.7%)	9 (5.1%)	8 (4.5%)	178 (100%)

An immunoassay ID-Heparin/PF4 PaGIA was performed in 40 samples of 40 patients with low probability of HIT. 34 (85%) plasma were negative, and 1 (2.5%) was positive, but not confirmed (HIPA- ELISA + ). 5 (12.5%) plasma were doubtful, for which no positive result have been confirmed (Table 4).

**Table 4:** correlation of The 4 T's scoring system and results of HIT antibody testing

4T's score	Patients HIT +	Patients HIT -	Patientsundetermined HIT	Total
Low	0 (0)	34(85)	6(15)	40 (22.5%)
Intermediate	10 (7.7)	100 (77.5)	19 (14,8)	129 (72.5%)
High	2 (22,2)	5 (55.5)	2 (22.2)	9 (5.1%)
Total	12	139	27	178

Our results shown that in low probability group, 85% of plasma were found negative versus 55.5% in the high probability group. Conversely, 22.2% of patients were HIT positive in high probability group versus 0% in the low probability group. In the main group of intermediate score, 7.7% of patients were found positive and 77.5% negative.

For patients with low risk of HIT, when the research of anti PF4 by ID-heparin/PF4 PaGIA was negative or not confirmed by both ELISA and HIPA, the therapeutic decision was to continue treatment with heparin. This was the case in 40 patients (22.5%). For patients with an intermediate risk, the rule was stopping of heparin and initiating of dapanoroide prophylactic dose, in the absence of thrombosis, or curative dose in case of arterial or venous thrombosis. One patient was classified as intermediate risk and presented an extension of deep venous thrombosis with heparin; he was put under dapanaroide curative doses at once. For patients with high risk (5.1%) the rule was always to stop heparin and substitute it by the danaparoid curative dose.

## Discussion

HIT must be identified as early as possible, because the lack of appropriate and early treatment may expose a risk of dramatic complications which can be life-threatening. In several patients, clinical criteria are not sufficient for the diagnosis of HIT and laboratory assays demonstrating the presence of heparin-dependant antibodies are essential. Recently, several reports have suggested the usefulness of the clinical pretest probability scoring system, the 4 T's score. We evaluated the efficacy of this score in our biological diagnostic strategy.

A large majority of patients recruited in our study had an intermediate risk of HIT (72.5%), this was also the case in the study reported by Lo and al [14]. The strong proportion of internal medicine patients and intensive care patients (62.2%) who presented various associated pathologies and who received several drugs inducing thrombocytopenia could explain the majority of intermediate scores [12]. In this group, we observed the larger number of discrepancy between ELISA and HIPA.

Particle gel immunoassay has the advantage of providing a result in less than 1 hour after blood sampling. When it was the only test performed, negative results were found in 85%. But 15% could be considered as false positive because a positive result in both functional and immunological assays was not confirmed.

None of the 40 patients who had a low clinical score tested positive for clinically significant HIT antibodies. This indicates that the clinical score has the potentially useful property of predicting which patients are most unlikely to have a serological profile indicating the presence of HIT [12, 15].

These results confirmed that the negative predictive value (NPV) of the HIT score was high. This high NPV of the 4Ts score was reported by other teams who had applied this scoring system [12, 14].

Ten (7.7%) patients with an intermediate clinical score tested positive for clinically significant HIT antibodies. Two (22.2%) patients with a high score tested positive for clinically significant HIT antibodies. Our results are in agreement with the literature data (Table 5) which show that the negative predictive value of the 4TS score is high and may therefore be useful to exclude HIT in case of low probability of the pretest. [12, 14, 16, 17]. Unlikely, the positive predictive value of the test is generally low and sometimes differs considerably from one center to another. This difference may be related to several factors such as the clinical experience of the physicians applying the scoring system; differences in the frequency of HIT; and it's also possible that various methods of assessment used by different centers, could explain the part of the differences in the obtained data. This fact shows that a better assessment of this score requires more studies.

**Table 5:** Characteristics of some studies evaluating the score 4Ts

	Lo GK and all	Lo GK and all	Pouplard and all	Strutt and al	Crowther and al	Our series
Sample size	100	236	213	80	50	178
**Strengths** : Type of study	Prospective	Prospective	Prospective, multicentre	Prospective	Prospective	Prospective
Category of patients	Medical and surgical inpatients	Medical and surgical inpatients	Medical and surgical inpatients	Medical and surgical inpatients		Medical and surgical inpatients
Technical assessment	SRA and EIA	HIPA andEIA	SRA		SRA	HIPA and ELISA
**Weakness**				Only ELISA	Only medical and chirurgical intensive care	
NPV of low clinical score 4Ts	98,4%	100%	100%	91%	100%	85%
PPV of intermediate clinical score	28,6%	7,9%	10,9%	NR	NR	7,7%
PPV of high clinical score 4Ts	100%	21,4%	80%	NR	NR	22,2%

EIA: enzyme immune assay; SRA: serotonin release assay; HIPA: heparin-induced platelet activation assay

Our results show that the higher the clinical score, the higher the number at positive results observed. The converse is also true. Furthermore, discrepancies were noted between clinical situations strongly suspecting HIT and negative test results, as 5 (55.5%) patients with a high score tested negative for clinically significant HIT antibodies. The specificity of the immunological and functional test is high only in a context suggestive of HIT. Both methods should be considered complementary in the diagnostic strategy.

## Conclusion

Our results were consistent with those of previous studies, confirming the usefulness of the clinical scoring system for the diagnosis of HIT. However, the clinical relevance of the 4T score must be validated on a larger scale and it does not seem suitable for all clinical situations. Close contact between clinicians and specialists in homeostasis is essential to the relevance of the diagnosis and to optimize the management of this syndrome.
